# Mr. Mortimer's Official Report to Dr. Dickson, Physician to the Fleet, Respecting the Fever in Marie Galante in 1808
*It is unnecessary to state the reasons why this paper has not previously appeared; nor the source whence we have derived it. We think it worthy of insertion, among the many documents which we have industriously collected on this interesting subject. Editors.


**Published:** 1817-07

**Authors:** John Mortimer

**Affiliations:** Surgeon. To Dr. Dickson, Physician to the Fleet, &c. &c. &c.


### *Mr.* Mortimer's *Official Report to Dr.* Dickson, *Physician to the Fleet, respecting the Fever in Marie Galante in* 1808.*

Sir, Marine Garrison, Marie Galante, August 8, 1808.

I Sit down, after a short residence, to inform you of my proceedings; and unsuccessful as they have been, I hope

---

* It is unnecessary to state the reasons why this paper has not previously you will give me credit for every exertion to resist the progress of disease, and, where that has failed, to smooth the passage to that bourne whither so many of our fellow-creatures are gone.

My first endeavour, on undertaking this arduous situation, was to make clean the apartments occupied, and to select others where disease had not yet appeared. You, also, engaged in this; and, after much inquiry, fixed on one at the windward extremity of the Savannah; and another high up in the country. To the last, every exertion was used by myself and assistants to send as many men as conveyances could be procured for. To the house also, below (occupied by a few previous to our arrival), I sent as many as possible.

For the first two or three days, such was the amendment produced by the measures you concerted, that I was enabled to remove fifty convalescents.

The extreme ruggedness of the road, and poverty of the cattle, could not have escaped your observation: these, together with heavy rains, obliged us to desist, until the strength of our cattle was recruited. The removal, however, of fifty men gave me a full opportunity of scouring out and ventilating the wards; of separating the better from the bad; the ulcers from the fevers; and those of different malignity to a distance from each other. By such precautions, carried into execution with a promptitude and zeal which nothing but the strongest conviction of the good expected to be derived from them could have actuated, I calculated, at least, on the remission of disease. I had drawn to the assistance of the sick an additional number of nurses; and had received unlimited powers from the Governor to apply all the means which time and practice might suggest for the amelioration of their condition. To account for the causes of disease, would be a task too difficult and presumptuous for me to enter on. The three following, however, appear to have had their influence separate and combined:

1st. The period of the year.
2d. Intemperance.
3d. Sedative effects of fear.

I have been particular in my inquiries amongst some

---

appeared; nor the source whence we have derived it. We think it worthy of insertion, among the many documents which we have industriously collected on this interesting subject. EDITORS.

well informed inhabitants (who talk English, and are well affected towards us) as to the general state of health of the people of this island, and the supposed causes of its derangement.

For three months only in the year do the inhabitants generally enjoy good health. These are, *April, May, and June.* From July to September (the worst of the whole), mortality prevails, and often to an alarming extent. In these months rain falls in torrents; and as they are often succeeded by intense heat, exhalations from pools, marshes, and every stagnant water, follow, and are considered as the chief sources of disease.

The aspect of the country seems particularly favourable to such exhalations. On viewing it, you almost constantly find hills of easy ascent, intersected by lesser declivities, and these, on both sides, encompassed by swamps; so that, whether in the interior or the town, sickness nearly equally obtains.

The diseases arising from these causes are, the continued, becoming what they term the low putrid, fever; inveterate intermittents; and remittents. They continue, altering their type according to the severity of the season, and varying susceptibility of the human frame.

If such changes in the seasons can be established as sufficient causes for the production of fevers amongst the inhabitants, may we not, without any great stretch, apply them, in greater force, to our garrison? The same quantity of cause produces different degrees of effect, according to the susceptibility at the time of seizure.

That our men are more liable to disease need not be wondered at, if we consider their mode of living. — The inhabitants most studiously avoid all salted animal food, during the heat of the day; live on fish and different sour crouts; and before sun-rise, and after sun-set, indulge heartily in the pleasures of the table.

How splendid is a Frenchman's table, when decorated with the various fruits of the earth — with fish, and perhaps with fowl! This, though partly habit, is more agreeable, by the belief that it renders them more fit for the duties of their station; and they fail not to recommend it to us, as the surest means of lessening inflammatory action, and consequently counteracting disease. I have myself particularly followed their judicious recommendation, and have failed not to inculcate its utility on the minds of others under my influence.

Instead of the common diet of salt meat and bread, half the quantity of meat was generally exchanged with the inhabitants for a sufficiency of vegetables, both for soup and their meat. This barter, salutary as it was in itself, in time led to the same practice for new rum; and I have no doubt had its share in producing the great mortality among the troops. Scarce a morning passed away that men were not confined for drunkenness! From the frequent suddenness of attack after this state, I believe drunkenness to have been a chief source of fever.

Its mode of attack was very different, appearing greatly to depend on previous habits of life. Those who were long known to have freely indulged themselves with new rum, were almost invariably seized with vomiting; generally less pain in the head than the more plethoric, but more in the loins and legs; and though less arterial action was perceptible, debility succeeded with great rapidity; and those who were not relieved in twenty-four hours, most generally died, throwing up, to the last, large quantities of atrabilious fluid.

The common mode of seizure was generally as follows: A day or two previous to the attack, the patient lost his spirits and appetite; and if, at this period, his eyes were examined, their lustre was diminished, and the vessels of the tunica conjunctiva replete with brown red blood. This state was soon followed by an acute pain in the forehead, seldom extending to the temples or back part. If no relief was here afforded, retching and vomiting came on; heat of skin was remarkably increased; and the pains in the back and calves of the legs became very distressing. The belly was generally costive. So far as my observation went, if these symptoms continued without an alleviation of vomiting (the most distressing and dangerous symptom of all) beyond thirty hours, the patient almost certainly died.

I have now described (I fear with insufficient accuracy) the pathognomonic symptoms of this fever. Of its mode of cure, much cannot be expected from me, when so many have failed before. Forgive me, nevertheless, when I express my faith in blood-letting, and think it has a claim to practical attention, beyond any other remedy now in use.

I am indebted to copious blood-letting, at the commencement of my illness, for my life; and I practised it under the same favourable circumstance with a great many others, and they did well.

The commencement of the disease is inflammatory; and though, towards its termination, it assumes the highest form of typhus gravior; yet may not this be in some degree attributed to the very hurried circulation which, in the worst cases, invariably obtains? And if so, have we so certain and sudden a mean, of arresting arterial action?

With great respect,
I am, Sir,
Your obedient and very humble servant,
JOHN MORTIMER, Surgeon.

To Dr. Dickson,
Physician to the Fleet,
&c. &c. &c.

